# CO_2_ Transport by PIP2 Aquaporins of Barley

**DOI:** 10.1093/pcp/pcu003

**Published:** 2014-01-30

**Authors:** Izumi C. Mori, Jiye Rhee, Mineo Shibasaka, Shizuka Sasano, Toshiyuki Kaneko, Tomoaki Horie, Maki Katsuhara

**Affiliations:** ^1^Institute of Plant Science and Resources, Okayama University, 2-20-1 Chuo, Kurashiki, 710-0046 Japan; ^2^Division of Applied Biology, Faculty of Textile Science and Technology, Shinshu University, 3-15-1, Tokida, Ueda, Nagano, 386-8567 Japan; ^3^Present address: Faculty of Sciences, University of South Bohemia in Ceske Budejovice, Czech Republic.; ^4^Present address: Department of Cardiovascular Physiology, Graduate School of Medicine, Dentistry and Pharmaceutical Sciences, Okayama University, 2-5-1, Shikata-cho, Kita-ku, Okayama, 700-8558 Japan.

**Keywords:** Aquaporin, Barley, Carbon dioxide, Plasma membrane intrinsic protein 2

## Abstract

CO_2_ permeability of plasma membrane intrinsic protein 2 (PIP2) aquaporins of *Hordeum vulgare* L. was investigated. Five PIP2 members were heterologously expressed in *Xenopus laevis* oocytes. CO_2_ permeability was determined by decrease of cytosolic pH in CO_2_-enriched buffer using a hydrogen ion-selective microelectrode. HvPIP2;1, HvPIP2;2, HvPIP2;3 and HvPIP2;5 facilitated CO_2_ transport across the oocyte cell membrane. However, HvPIP2;4 that is highly homologous to HvPIP2;3 did not. The isoleucine residue at position 254 of HvPIP2;3 was conserved in PIP2 aquaporins of barley, except HvPIP2;4, which possesses methionine instead. CO_2_ permeability was lost by the substitution of the Ile254 of HvPIP2;3 by methionine, while water permeability was not affected. These results suggest that PIP2 aquaporins are permeable to CO_2_. and the conserved isoleucine at the end of the E-loop is crucial for CO_2_ selectivity.

Sequence data from the article can be found in the DNA Data Bank Japan (DDBJ) data library under the following accession numbers: *HvPIP2;1*, AB219366; *HvPIP2;2*, AB377269; *HvPIP2;3*, AB275280; *HvPIP2;4*, AB219525; and *HvPIP2;5*, AB377270, respectively.

## Introduction

It is widely accepted that carbon dioxide is transported across biomembranes through aquaporins in a cell (for a review, see Kaldenhoff, 2012). Plant aquaporins are classified into five subfamilies: plasma membrane intrinsic proteins (PIPs), tonoplast intrinsic proteins (TIPs), nodulin 26-like intrinsic proteins (NIPs), small basic intrinsic proteins (SIPs) and X intrinsic proteins (XIPs) (Danielson and Johanson 2008, Maurel et al. 2008). PIP aquaporins consist of two major subgroups, PIP1 and PIP2. In general, PIP2s have a higher capacity to facilitate water transport in heterologous expression systems, while PIP1s show low or no water transport activity (Chaumont et al. 2000, Johanson et al. 2001, Moshelion et al. 2002, Horie et al. 2011, Shibasaka et al. 2012). The tobacco PIP1 aquaporin, NtAQP1, displayed CO_2_ transport activity in *Xenopus laevis* oocytes (Uehlein et al. 2003). NtAQP1 facilitated CO_2_ transport when expressed in yeast cells, but the tobacco PIP2, NtPIP2;1 did not (Otto et al. 2010). The Arabidopsis PIP1, AtPIP1;2, was also shown to be permeable to CO_2_, while the PIP2, AtPIP2;3, was not, in a yeast expression system (Heckwolf et al. 2011). The involvement of NtAQP1 in mesophyll CO_2_ conductance was demonstrated by overexpression and RNA interference (RNAi) suppression experiments (Flexas et al. 2006, Uehlein et al. 2008). The knockout mutant of the *AtPIP1;2* gene exhibited a decreased mesophyll conductance, indicating that AtPIP1;2 facilitates the diffusion of CO_2_ in leaves (Uehlein et al. 2012a). Ectopic expression of the ice plant PIP1, *McMIPB*, in tobacco plants increased the CO_2_ assimilation rate and mesophyll CO_2_ conductance (Kawase et al. 2013). These reports imply a physiological significance of the CO_2_ permeation of PIP1 aquaporins. On the other hand, Hanba et al. (2004) showed that overexpression of the barley PIP2, HvPIP2;1, in rice plants increased their photosynthetic rate and mesophyll CO_2_ conductance. This suggested that some PIP2 aquaporins are possibly permeable to CO_2_. Recently, the CO_2_ permeability of NtPIP2;1 was shown by means of polymer-embedding experiments (Uehlein et al. 2012b), although the CO_2_ permeability of NtPIP2;1 was not detected in the heterologous expression system (Otto et al. 2010). CO_2_ permeability of PIP2s has not been assessed in detail. In this study, we examined the CO_2_ permeability of five barley PIP2 aquaporins by assessing the pH decrease of the cytosol of *X. laevis* oocytes heterologously expressing HvPIP2s in CO_2_-enriched buffer. In addition, we determined a crucial amino acid for CO_2_ permeation by comparing the deduced amino acids of two closely related PIP2 aquaporins, HvPIP2;3 and HvPIP2;4.

## Results

### CO_2_ permeability of barley PIP2s

The CO_2_ permeability of *HvPIP2* cRNA-injected *Xenopus laevis* oocytes was investigated by measuring the rate of cytosolic acidification in CO_2_-enriched buffer (Nakhoul et al. 1998). Cytosolic pH was measured by means of a hydrogen ion-selective microelectrode. As they could not be quantified separately, the portion of CO_2_ and H_2_CO_3_ in the solution is designated as CO_2_/H_2_CO_3_ herein.

**Fig. 1:**
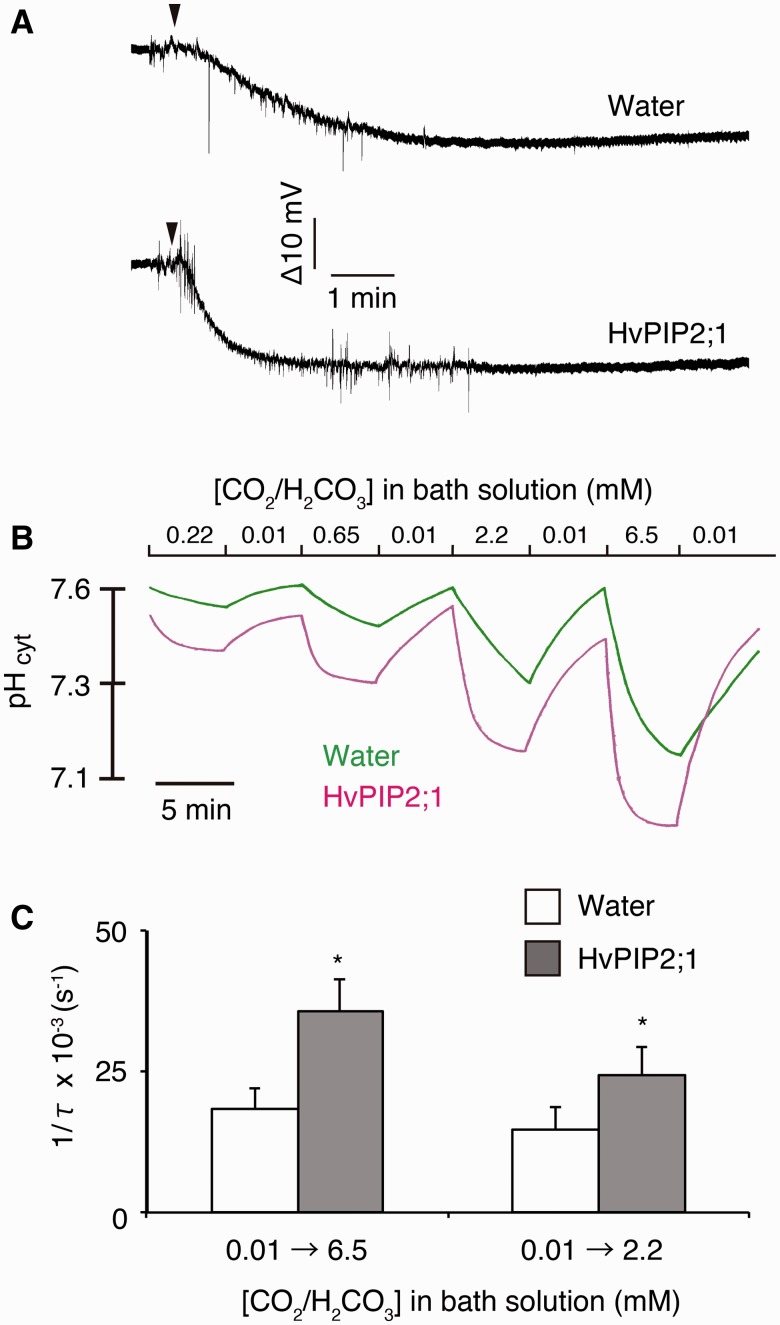
Cytosolic acidification of *HvPIP2;1*-injected *X. laevis* oocytes induced by perfusion of carbon dioxide-enriched buffer. (A) Typical raw recordings of water-injected and *HvPIP2;1* cRNA-injected oocytes by perfusion with modified Barth’s solution, of which the NaCl and NaHCO_3_ concentrations and pH were modified. The raw recordings represent the difference of the reading of two electrodes (V_pH_ – V_ref_). V_pH_ and V_ref_ indicate the voltage reading of the hydrogen ion-selective microelectrode and the membrane potential microelectrode, respectively. The pH of the buffer was adjusted to 7.31, so that the ratio of CO_2_/H_2_CO_3_ to 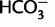 was 0.1. The concentration of CO_2_/H_2_CO_3_ was changed from 0.1 mM to 6.5 mM by perfusion. The perfusion was initiated where indicated by arrowheads. The buffer around the oocyte was replaced 10 s after the start of the perfusion in a typical measurement. This duration was estimation by pH change without an oocyte present. (B) The cytosolic pH change of water-injected (Water, green line) and *HvPIP2;1* cRNA-injected (HvPIP2;1, magenta line) oocytes by perfusion with modified Barth’s solution, of which the NaCl and NaHCO_3_ concentrations and pH were modified. The cytosolic pH was measured by hydrogen ion-selective microelectrodes. In the bath solution with 0.01 mM CO_2_/H_2_CO_3_, NaHCO_3_ was substituted with NaCl and the concentration of CO_2_/H_2_CO_3_ was determined by equilibration with the ambient air. The bath solutions which included 0.22, 0.65, 2.2 and 6.5 mM CO_2_/H_2_CO_3_ (CO_2_-enriched buffer) were prepared by replacing NaCl with NaHCO_3_ to give the appropriate CO_2_/H_2_CO_3_ concentrations in the bath solution. The CO_2_-enriched buffers were aliquoted and sealed with caps immediately after the preparation to prevent the diffusional loss of CO_2_ gas into the air. The oocytes were equilibrated to the bath solution containing 0.01 mM CO_2_/H_2_CO_3_ and impaled with the microelectrodes as described in the Materials and Methods. Subsequently, the bath solution was perfused with a peristaltic pump at a rate of 400 µl min^−1^. Hum noise (60 Hz) was cancelled in silico. Note that the *y*-axis was converted from electric potential to pH according to calibration lines. A typical calibration line is shown in Supplementary Fig. S6. (C) Rate of cytosolic acidification of water-injected (Water) and *HvPIP2;1* cRNA-injected (HvPIP2;1) oocytes as shown by the reciprocal of the time constant (1/τ). The CO_2_/H_2_CO_3_ concentration in the bath solution was replaced by perfusion (400 µl min^−1^) from 0.01 mM to 6.5 mM (0.01→6.5) or 2.2 mM (0.01→2.2). τ was determined by exponential curve fitting. Water (0.01→6.5), *n* = 11. HvPIP2;1 (0.01→6.5, *n* = 10. Water (0.01→2.2), *n* = 7. HvPIP2;1 (0.01→2.2), *n* = 6. Asterisks indicate a significant difference of the mean of HvPIP2;1 from that of the water control at α = 0.05.

The difference in electric potentials between the hydrogen ion-selective microelectrode and the membrane potential microelectrode was decreased by the replacement of the buffer containing 0.01 mM CO_2_/H_2_CO_3_ in water-injected and *HvPIP2;1* cRNA-injected oocytes (Fig. 1A). This indicates that the cytosol of oocytes was acidified by perfusion with the CO_2_-enriched buffer. The rate of acidification was higher in *HvPIP2;1* cRNA-injected oocytes. The cytosolic pH of water-injected and *HvPIP2;1* cRNA-injected oocytes was approximately 7.5–7.6 in the bath solution containing 0.01 mM CO_2_/H_2_CO_3_ (Fig. 1B). The cytosolic pH was gradually acidified by replacing the bath solution with 0.22 mM CO_2_/H_2_CO_3_ in the water-injected oocytes. The lowered cytosolic pH returned to its former high value by perfusion of the bath solution back to 0.01 mM CO_2_/H_2_CO_3_. The cytosolic pH change was repeatable and the acidification was enhanced along with increasing concentrations of CO_2_/H_2_CO_3_ (0.65, 2.2 and 6.5 mM) (Fig. 1B). In *HvPIP2;1* cRNA-injected oocytes, cytosolic pH changed to the same orientation. However, the acidification rate was apparently more rapid than with water-injected oocytes (Fig. 1B). The cytosolic pH reached a plateau within 5 min in *HvPIP2;1* cRNA-injected oocyte in the typical experiments. As the acidification of the cytosol by CO_2_-enriched buffers apparently followed an exponential curve, the time constant (τ) of the acidification was determined by exponential curve fitting. The reciprocal of τ (1/τ) was significantly higher in *HvPIP2;1* cRNA-injected oocytes compared with the water-injected controls, regardless of the CO_2_/H_2_CO_3_ concentration (2.2 and 6.5 mM) (Fig. 1C). This indicates that CO_2_ or H_2_CO_3_ in the bath solution migrated across the cell membrane into the frog oocytes via HvPIP2;1 and immediately dissociated to H^+^ and 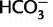 in the cell to acidify the cytosol.

**Fig. 2:**
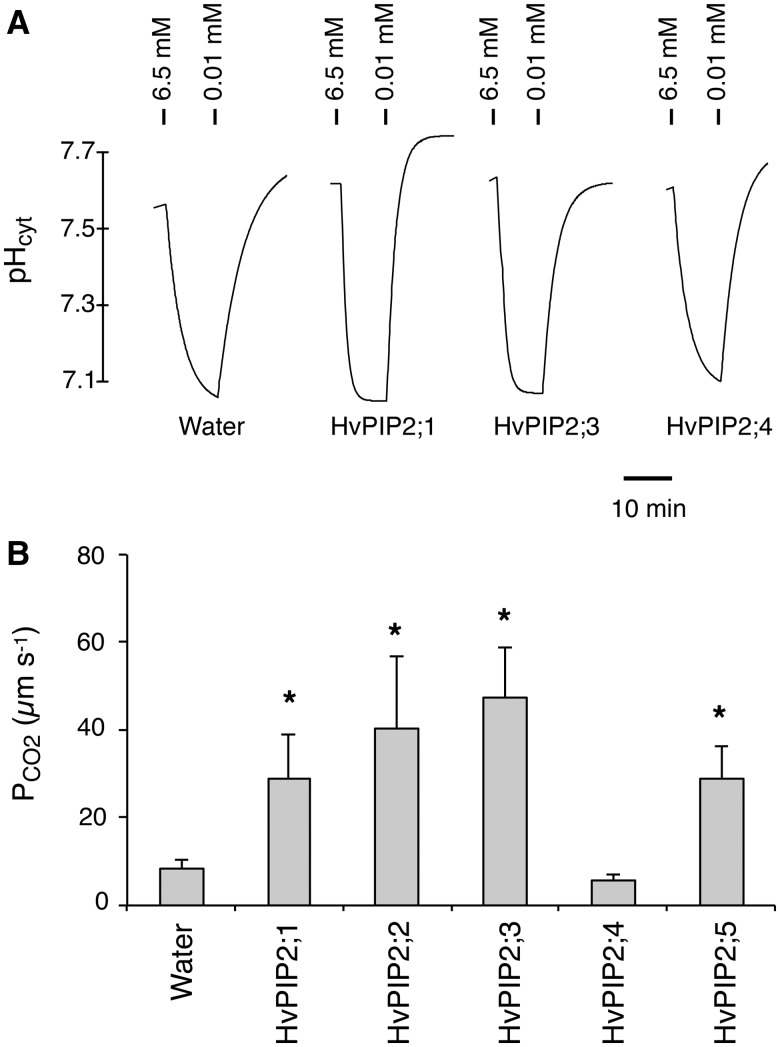
CO_2_ permeability of PIP2 aquaporins of barley. (A) Representative traces of cytosolic pH change of water-, *HvPIP2;1* cRNA-, *HvPIP2;3* cRNA- and *HvPIP2;4* cRNA-injected *X. laevis* oocytes. cRNAs and carbonic anhydrase were injected 24–48 h before the measurements. Ticks above the traces indicate where the bath solutions were replaced with the modified Barth’s solution containing the designated concentrations of CO_2_/H_2_CO_3_. (B) P_CO2_ of the cell membrane of *X. laevis* oocytes injected with water (*n* = 14), *HvPIP2;1* cRNA (*n* = 10), *HvPIP2;2* cRNA (*n* = 5), *HvPIP2;3* cRNA (*n* = 6), *HvPIP2;4* cRNA (*n* = 7) or *HvPIP2;5* cRNA (*n* = 3). Error bars indicate the SEM. Asterisks indicate significant difference of the mean from the water-injected control (Water) by Student’s *t*-test at α = 0.05.

In addition to HvPIP2;1, we examined the CO_2_ permeability of the other four PIP2 members identified from barley (Horie et al. 2011) to gain insight into the CO_2_ transport of PIP2 aquaporins. The cytosolic pH of *X. laevis* oocytes was 7.5–7.6 in the bath solution containing 0.01 mM CO_2_/H_2_CO_3_ (Fig. 2A). Upon replacement of the bath solution with CO_2_-enriched buffer (6.5 mM CO_2_/H_2_CO_3_), the cytosolic pH decreased promptly toward 7.0–7.1 in *HvPIP2;3* cRNA-injected as well as *HvPIP2;1* cRNA-injected oocytes (Fig. 2A). In *HvPIP2;4* cRNA-injected oocytes, the pH decrease was not as fast as in *HvPIP2;1* and *HvPIP2;3* cRNA-injected oocytes (Fig. 2A). The acidified pH of the oocytes returned by replacement of the buffer with the low CO_2_ buffer (0.01 mM CO_2_/H_2_CO_3_). The CO_2_ permeability (P_CO2_) of the cell membrane of oocytes was calculated from the time constant, final cytosolic pH after the acidification and surface to volume ratio of oocytes (Yang et al. 2000) (Fig. 2B). The P_CO2_ values of the water-injected oocytes and *HvPIP2;4* cRNA-injected oocytes were low and no significant difference was observed between the two. On the other hand, the oocytes injected with cRNAs of *HvPIP2;1*, *HvPIP2;2*, *HvPIP2;3* and *HvPIP2;5* demonstrated increased P_CO2_ (3- to 5-fold). This strongly suggests that a subset of barley PIP2s, HvPIP2;1, HvPIP2;2, HvPIP2;3 and HvPIP2;5, are CO_2_ permeable in the heterologous expression system in *X. laevis* oocytes. Our previous report demonstrated that all five HvPIP2s were permeable to water when expressed in oocytes (figs. 4 and 5 in Horie et al. 2011).

### Isoleucine 254 (I-254) is an important factor for CO_2_ permeability of HvPIP2;3

**Fig. 3:**
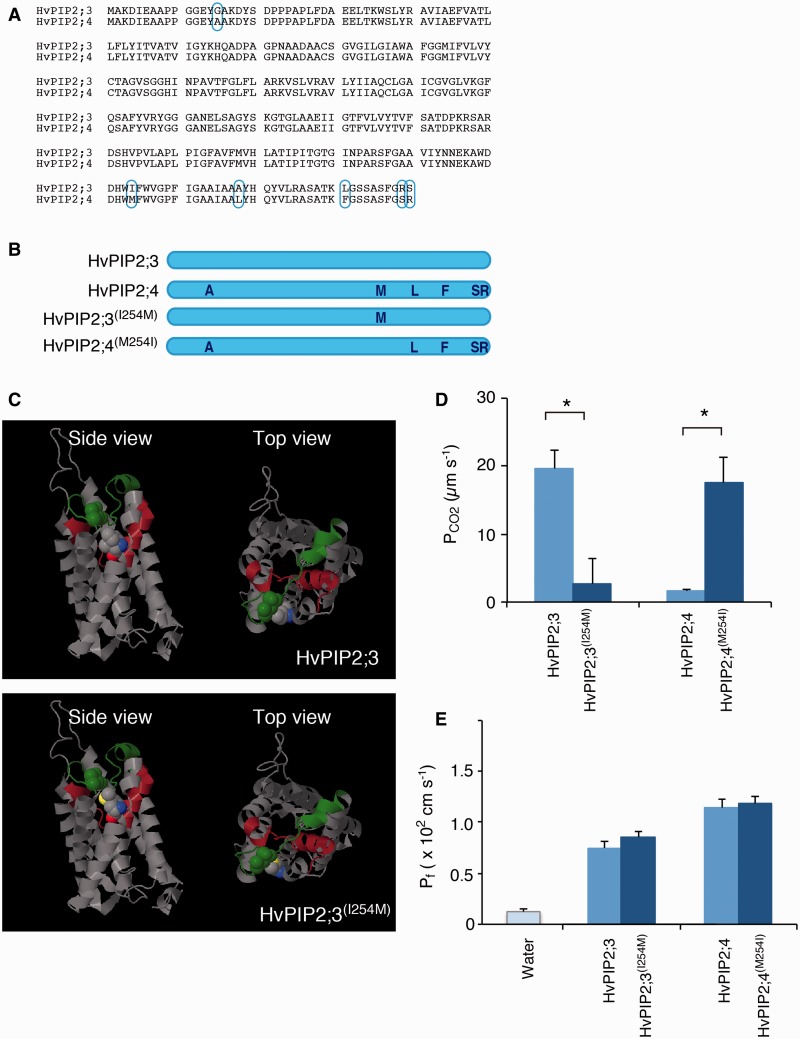
Isoleucine 254 of HvPIP2;3 is one of the key factors determining CO_2_ permeability. (A) Alignment of amino acid sequences of HvPIP2;3 and HvPIP2;4. Six different amino acids are designated by cyan ellipsoids. (B) Illustrated representation of amino acid substitution constructs. Indigo letters indicate the amino acids of HvPIP2;4 origin; the remainder are those of HvPIP2;3. (C) Three-dimensional homology modeling of HvPIP2;3 and HvPIP2;3^(I254M)^ molecules. The model was constructed based on an X-ray diffraction structural model of spinach SoPIP2;1. The yellow ball shape indicates the sulfur atom of M-254. Red and blue ball shapes indicate oxygen and nitrogen atoms, respectively, of the 254th amino acid. The green ball shape indicates L-165 close to M-254. The green stretch indicates the C-loop. The brown strand and helix indicate the E-loop. (D) CO_2_ permeability (P_CO2_) of the cell membrane of *X. laevis* oocytes injected with HvPIP2;3 (*n* = 6), HvPIP2;4 (*n* = 3) and the amino acid-swapped constructs, HvPIP2;3^(I254M)^ (*n* = 7) and HvPIP2;4^(M254I)^ (*n* = 3). cRNAs and carbonic anhydrase were injected 24–48 h before the measurements. Error bars indicate the SEM. Asterisks indicate a significant difference of the mean (Student’s *t*-test, α = 0.05). (E) Osmotic water permeability (P_f_) of the cell membrane of *X. laevis* oocytes injected with water (*n* = 8), *HvPIP2;3* cRNA (*n* = 9), *HvPIP2;3^(I254M)^* cRNA (*n* = 9), *HvPIP2;4* cRNA (*n* = 9) and *HvPIP2;4^(M254I)^* cRNA (*n* = 10). Error bars indicate the SEM. No significant difference was observed between HvPIP2;3 and HvPIP2;3^(I254M)^, or between HvPIP2;4 and HvPIP2;4^(M254I)^ (Student’s *t*-test, α = 0.05).

The amino acid identity between HvPIP2;3 and HvPIP2;4 is very high, and only six amino acids are different out of 296 (Fig. 3A). However, the results described above suggested that HvPIP2;3 was permeable to CO_2_/H_2_CO_3_, but HvPIP2;4 apparently was not. We examined the CO_2_ permeability of the amino acid-substituted aquaporins of HvPIP2;3 and HvPIP2;4 (Fig. 3B) to determine the structural basis of the difference in CO_2_ permeability between the two aquaporins.

Simultaneous substitution of four amino acids of HvPIP2;3, A-268, L-281, R-289 and S-290, to L-268, F-281, S-289 and R-290 [HvPIP2;3^(LFSR)^] did not affect the acidification of the cytosol of *HvPIP2;3^(LFSR)^* cRNA-injected oocytes compared with *HvPIP2;3*-injected oocytes (Supplementary Fig. S1B). The osmotic water permeability (P_f_) of HvPIP2;3 and HvPIP2;3^(LFSR)^ was not significantly different (*P* > 0.05, Supplementary Fig. S1C). This indicates that the four amino acids in the C-terminal stretch are not involved in the CO_2_ permeability of HvPIP2;3. Substitution of I-254 of HvPIP2;3, which is localized at the edge of the E-loop, by methionine (Fig. 3C) resulted in substantial repression of the P_CO2_ (Fig. 3D). Substitution of methionine 254 (M-254) of HvPIP2;4 by isoleucine, HvPIP2;4^(M254I)^ (Fig. 3B) caused activation of P_CO2_ (Fig. 3D). The P_f_ of HvPIP2;3^(I254M)^ and HvPIP2;4^(M254I)^ was examined to test whether the mutated aquaporins were functional (Fig. 3E). The substitution of I-254 of HvPIP2;3 and M-254 of HvPIP2;4 did not show any apparent effect on P_f_. This indicates that I-254 of HvPIP2;3 is crucial to the CO_2_ permeability.

## Discussion

We provide evidence that barley PIP2 aquaporins, HvPIP2;1, HvPIP2;2, HvPIP2;3 and HvPIP2;5, but not HvPIP2;4, facilitate CO_2_ transport across biomembranes (Figs. 1, 2). The CO_2_ permeability of PIP1 aquaporins has been demonstrated using heterologous expression systems. The CO_2_ permeability of NtAQP1 was examined in a *Saccharomyces cerevisiae* expression system and a *X. laevis* oocyte expression system (Uehlein et al. 2003, Heckwolf et al. 2011). The CO_2_ permeability of AtPIP1;2 was shown in a yeast expression system. These expression systems demonstrated that PIP2 aquaporins neither NtPIP2;1 or AtPIP2;3 did not facilitate transport of CO_2_ (Otto et al. 2010, Heckwolf et al. 2011). Meanwhile, Uehlein et al. (2012b) demonstrated the CO_2_ permeability of NtPIP2;1 by embedding the isolated protein in a polymer membrane. The discrepancy in CO_2_ permeability of NtPIP2;1 described by Otto et al. (2011) and Uehlein et al. (2012b) was discussed in terms of the biological membrane having a certain background CO_2_ permeability (Uehlein et al. 2012b). Hanba et al. (2004) suggested that HvPIP2;1 is permeable to CO_2_ by using overexpression in plants. However, direct evidence for the CO_2_ permeability of PIP2s had not been provided previously. In this study, we successfully demonstrated the CO_2_ permeability of barley PIP2s in a *X. laevis* oocyte expression system. Surprisingly, our data suggested that four out of five known PIP2s from barley were permeable to CO_2_. More PIP2s might be CO_2_ permeable than we expected.

In this study, we examined the CO_2_ permeability of only PIP2s of barley. The present work does not deny the possibility that PIP1s might be permeable to CO_2_ , as HvPIP1s were not examined. It was previously shown that tobacco NtAQP1 localized on the cell membrane of the oocytes and was permeable to CO_2_ (Uehlein et al. 2003). However, for barley PIP1s, we did not observe efficient localization to the cell membrane (Supplementary Results; Supplementary Fig. S2). This corresponds to the lack of water transport activity of HvPIP1;2 (Horie et al. 2011). One of the reasons for the undetected water permeability of HvPIP1s in the previous study (Horie et al. 2011) may be the low efficiency of targeting of PIP1s to the oocytes’ cell membrane. For this reason, we did not examine the CO_2_ permeability of PIP1s of barley in this study. The permeability of HvPIP1s to CO_2_ remains an open question.

It was suggested in this study that HvPIP2;3 is permeable to CO_2_ but HvPIP2;4 is not, although only six amino acids are different (Fig. 2). Taking advantage of this difference, we determined the amino acids essential for CO_2_ permeability. We identified I-254 of HvPIP2;3 as a critical amino acid residue (Fig. 3; Supplementary Fig. S1). The substitution of I-254 by methionine substantially impaired CO_2_ permeability. However, the P_f_ was not lost (Fig. 3E).

Zhang et al. (2010) reported that valine/isoleucine (V/I) in the membrane-spanning helix 2 of the rice PIP2s was crucial for water permeation activity. The amino acid corresponding to this position in OsPIP1s is alanine. The substitution of V/I by alanine decreased the P_f_ (Zhang et al. 2010). In HvPIP2;3 and HvPIP2;4, the corresponding position is V. This residue coincides with significant P_f_ of HvPIP2;3 and HvPIP2;4. Suga and Maeshima (2004) reported that the specific V residue in the E-loop of radish PIP2s was important for water transport. The substitution of V with I, as seen in radish PIP1s, substantially decreased the water permeability. The corresponding amino acid in HvPIP2;3 and HvPIP2;4 was V. There has been no previous report of finding a structural basis for the CO_2_ permeability of aquaporins.

I-254 is located at the C-terminal end of the E-loop. When substituted with methionine, this residue was predicted to be sited proximal to the oxygen molecule of the carboxyl group of the main chain close to leucine 165 in the C-loop, with the sulfur atom facing the C-loop (Fig. 3C). The sulfur atom of methionine interacts with the nucleophilic oxygen atom (Fig. 3C) (Chakrabarti and Bhattacharyya 2007). This interaction may result in a distortion of the C-loop and hamper the CO_2_ permeability of HvPIP2;3. Importantly, this isoleucine is highly conserved in PIP1 and PIP2 of barley, except HvPIP2;4 (Supplementary Fig. S3). The importance of isoleucine at the C-terminal end of the E-loop has not been examined previously. Recently an aquaporin from cyanobacterium was reported to be permeable to CO_2_ (Ding et al. 2013). The corresponding position was leucine instead of isoleucine in the algal aquaporin. If the mechanism of CO_2_ impermeability of the M-254-substituted aquaporin is attributed to the interaction with the C-loop via the sulfur atom, it can be easily understood why leucine did not disrupt the CO_2_ permeability. Supplementary Table S1 shows a list of PIP members whose CO_2_ permeability has been examined and the amino acid sequence at the C-terminal end of the E-loop. Supplementary Fig. S4 shows the alignment of amino acid sequences of PIP1s and PIP2s at the corresponding region of Arabidopsis, rice, maize and barley. It is noticeable that the V residue of AtPIP1;2 is capable of substituting for the I residue. NtPIP2;1 and AtPIP2;3 possess I at this position, but they were not permeable to CO_2_ in the heterologous expression systems (Otto et al. 2010, Heckwolf et al. 2011). These indicate that there are other factors for CO_2_ selectivity besides the specific amino acid residue at the C-terminal end of the E-loop. Comparative analyses of aquaporin polymorphism in the future will provide new insights with regard to CO_2_ permeability. It may lead to new technologies to regulate CO_2_ conductance in plants via aquaporins facilitating CO_2_ permeation.

## Materials and Methods

### Water transport activity assay in *Xenopus laevis* oocytes

The cDNAs of HvPIP2;3, HvPIP2;4, HvPIP2;3^(I254M)^ and HvPIP2;4^(M254I)^ were subcloned into the pXβGev1 expression plasmid vector, as reported previously (Horie et al. 2011). The plasmid was linearized with *Not*I, and capped cRNA was synthesized using the mMESSAGE mMACHINE T3 in vitro transcription kit (Ambion). Oocytes were isolated from adult female *X. laevis* and maintained as described previously (Katsuhara et al. 2002). Oocytes were injected with 50 nl of a cRNA solution containing 2 ng of RNAs 24–48 h before measurement. As a negative control, water-injected oocytes were used. The osmotic water permeability coefficient of oocytes was measured according to the procedures described previously (Katsuhara et al. 2002, Mahdieh et al. 2008).

### Construction of the hydrogen ion-selective microelectrode

A glass capillary with a filament (1.5 mm outer diameter/1.12 mm inner diameter, World Precision Instruments) was pulled with a micropipet puller (model P-1000, Sutter Instruments) under the conditions described in Supplementary Table S2. The glass pipet was then filled with a cocktail of hydrogen ionophore [1 : 5 : 4 mixture of hydrogen ionphore I cocktail A (Selectophore grade, Fluka), 0.5% polyvinyl chloride dissolved in tetrahydrofuran and tetrahydrofuran]. The glass pipet was left to stand for a day for the ionophore mixure at the tip of the pipet to solidify.

### CO_2_ permeability assay in *Xenopus laevis* oocytes

*Xenopus laevis* oocytes were prepared as described for the water transport activity assay (Horie et al. 2011). Each oocyte was injected with 25 ng of *HvPIP2;1*, *HvPIP2;2*, *HvPIP2;3*, *HvPIP2;4* or *HvPIP2;5* cRNA. A 25 ng aliquot of carbonic anhydrase (Catalog No. C-3934, Sigma-Aldrich) was simultaneously injected into the oocytes (Supplementary Results; Supplementary Fig. S5). The oocytes were impaled with a membrane potential microelectrode and a hydrogen ion-selective microelectrode. The membrane potential microelectrode was made in the same way as the hydrogen ion-selective microelectrode, except for filling with the ionophore cocktail; in this case, it was filled with 0.5 M KCl. The hydrogen ion-selective microelectrode was backfilled with the electrode solution containing 0.5 M KCl, 0.2 M MES and Tris (pH 6.0). The membrane potential microelectrode and hydrogen ion-selective microelectrode were attached to head stages (HS-9A and HS-2, respectively, Axon Instruments). The reference electrode was immersed in the bath solution via an agar bridge containing 3 M KCl. The dimensions of the chamber made of acrylic resin were 3 mm (depth) × 3 mm (width) × 25 mm (path length). The estimated time for solution exchange in the proximity of the oocyte with a flow rate of 400 µl min^−1^ was approximately 10 s. An amplifier, Axoclamp 900A (Axon Instruments), a digitizer, Digidata 1440A (Axon Instruments), and software, pCLAMP 10 (Axon Instruments), were used to acquire the voltage output of the microelectrodes. The recording rate was 1,000 Hz. The pH of the cytosol of the oocytes was determined from the difference between the two microelectrodes. The electrodes were calibrated with calibration solutions whose pH was buffered to 5.0, 5.5, 6.0, 6.5, 7.0 and 7.5 with either 0.1 M MES/Tris or PIPES/Tris, and contained 0.1 M KCl.

The P_CO2_ was determined according to a previous report (Yang et al. 2000). The final cytosolic pH was determined from the actual measurement at the end of the recordings. The time constant (τ) was obtained from the trace of the difference of the two microelectrodes by exponential curve fitting. The surface to volume ratio of oocytes was determined from the average of the diameter of 18 representative oocytes.

### Construction of HvPIP2;3^(I254M)^, HvPIP2;4^(M254I)^ and HvPIP2;3^(LFSR)^

Point mutation of the constructs for the amino acid substitution, HvPIP2;3^(I254M)^ and HvPIP2;4^(M254I)^, was done essentially according to a previous report (Zheng et al. 2004). The primers utilized are listed in Supplementary Table S3. cDNA of *HvPIP2;3^(LFSR)^* happened to be isolated naturally along with the cloning of the cDNA of HvPIP2;3. The corresponding sequence might exist in a natural population of *Hordeum vulgare* cv. Haruna nijyo to a minor extent.

### Homology modeling

Homology modeling was performed by the Workspace at the Swiss-Model website, URL: http://swissmodel.expasy.org/ (Arnold et al. 2006).

## Supplementary data

Supplementary data are available at PCP online.

## Funding

This work was supported The Nissan Science Foundation; a JSPS KAKENHI Grant-in-Aid for Scientific Research on Innovative Areas [grant No. 24114709 to I.C.M.]; the Ohara Foundation for Agricultural Research; the Program for Promotion of Basic Research Activities for Innovative Biosciences; Japan Science and Technology Agency (JST) [Adaptable and Seamless Technology Transfer Program through target-driven R&D, Exploratory Research, to M.K.]

## Supplementary Material

Supplementary Data

## Abbreviations

P_CO2_: CO_2_ permeability
P_f_: osmotic water permeability
PIP: plasma membrane intrinsic protein
