# Supplementary material for: CO2 Transport by PIP2 Aquaporins of Barley
Source: Plant Cell Physiol. 2014 Jan 30;55(2):251–7. doi: 10.1093/pcp/pcu003 (PMC3913445; doi:10.1093/pcp/pcu003)
Supplement: Supplementary Data [file supp_55_2_251__index.html]

CO2 Transport by PIP2 Aquaporins of Barley — CO2 Transport by PIP2 Aquaporins of Barley — CO2 Transport by PIP2 Aquaporins of Barley — Supplementary Data 

# CO2 Transport by PIP2 Aquaporins of Barley

## Supplementary Data

files

**Files in this Data Supplement:**

- Supplementary Data - pdf file
