# Supplementary material for: CO2 Transport by PIP2 Aquaporins of Barley
Source: Plant Cell Physiol. 2014 Jan 30;55(2):251–7. doi: 10.1093/pcp/pcu003 (PMC3913445; doi:10.1093/pcp/pcu003)
Supplement: Supplementary Data [file supp_pcu003_pcp-2013-e-00412-File005.pdf]

Supplementary Materials and Methods

Indirect immunofluorescence microscopy

Oocytes were injected with 50 ng of *HvPIP* cRNAs and incubated for 24 h at 18˚C. The oocytes were fixed in a 4 % (w/v) formaldehyde solution (pH 7.4) overnight. Fixed samples were embedded in 4% agarose. Oocytes were sliced (thickness: 50 µm) with a razor blade, then were treated with blocking solution (50 mM Tris pH 8.0, 150 mM NaCl, 0.1% Tween 20, 3% BSA) for 1 h at 25˚C. After blocking, anti-HvPIP1 rat antibody (generated against the synthetic peptide MEGKEEDVRLGANRY, Medical & Biological Laboratories Co., Japan) or anti-HvPIP2;1 antibody (Horie et al. 2011) was applied to samples for 1 h at 25˚C. After washing three times, the secondary antibodies (anti-rat IgG goat antibody conjugated with Alexa 647 [Invitrogen, Carlsbad, CA, USA] or anti-rabbit IgG goat antibody conjugated with Alexa 488 [Invitrogen, Carlsbad, CA, USA]) were applied for another 1 h. After washing twice with the TBS-T solution (50 mM Tris pH 8.0, 150 mM NaCl, 0.1% Tween 20) and once with TBS (50 mM Tris pH 8.0, 150 mM NaCl), samples were analyzed with a fluorescence microscope (BZ-8000, Keyence, Japan). Exposure time varied according to the fluorescence. Fluorescence intensity was measured along lines indicated in inset gray images (converted from color images) using WinRoof software (ver. 3.51, MITANI Corporation, 2000, Japan).

Supplementary Results

Barley PIP1 did not localize to cell membrane in *X. laevis* oocytes

Supplementary Fig. S2 shows that HvPIP1;2 did not localized in the cell membrane when *HvPIP1;2* cRNA was injected to the oocytes. Intensity of fluorescence of antibody against HvPIP2;1 was concentrated at the edge of the cell, namely in the cell membrane (Supplementary Fig. S2D). In contrast, the fluorescence of HvPIP1;2 was distributed evenly in the cell, indicating that HvPIP1;2 did not apparently accumulate in the cell membrane (Supplementary Fig. S2B). Essentially the same result was obtained for HvPIP1;4 (data not shown). Control oocytes injected with water showed virtually undetectable levels of the fluorescence (data not shown).

Twenty-five ng of carbonic anhydrase (CA) was enough for CO_2 permeability assay and PIP2s permeate CO_2, not H_2CO_3

It was reported that injection of carbonic anhydrase (CA) to the oocyte was essential for the measurement of CO_2 transport activity by cytosolic acidification (Nakhoul et al. 1998). They injected 50 ng CA per oocyte. However, the injection of 50 ng of CA was technically difficult, as it was viscous. Thus, we examined the effect of 25 ng CA on the CO_2 permeability (Supplementary Fig. S5). In the absence of CA, the acidification of oocytes induced by CO_2-enriched buffer was apparently delayed, regardless of injection of *HvPIP2;1* cRNA. Injection of 25 ng per oocyte CA together with *HvPIP2;1* cRNA dramatically increased the rate of acidification. The injection of 25 ng per oocyte yielded a comparably high $1/\tau$ to 50 ng per oocyte (Supplementary Fig. S5). Thus, we injected 25 ng CA per oocyte throughout this study.

CA catalyzes equilibration of CO_2 and H_2CO_3 in a water environment. It is not possible to chemically discriminate CO_2 and H_2CO_3 as a matter of fact. Which is the substrate of the aquaporins, CO_2 or H_2CO_3? The requirement of CA inside the cell suggests that the substrate is CO_2, rather than H_2CO_3. CO_2 migrating inside the cell

through the aquaporins will be converted to H_2CO_3 spontaneously by CA catalysis in the oocytes, which will in turn acidify the cytosol by dissociation to H^+ and bicarbonate ion.

Supplementary Figure legends

Supplementary Fig. S1. CO_2 permeability (P_{CO2}) of HvPIP2;3 with 4 amino acid substitutions at the carboxyl terminal stretch. (A) Illustrated presentation of HvPIP2;3$^{(LFSR)}$ construct. (B) P_{CO2} of *X. laevis* oocytes injected with water, *HvPIP2;3* cRNA and *HvPIP2;3$^{(LFSR)}$* cRNA. Error bar indicates standard error. Water, n = 5; HvPIP2;3, n = 5; HvPIP2;3$^{(LFSR)}$, n = 4. (C) P_f of *X. laevis* oocytes injected with water, *HvPIP2;3* cRNA and *HvPIP2;3$^{(LFSR)}$* cRNA. Error bar indicates standard error. Water, n = 8; HvPIP2;3, n = 9; HvPIP2;3$^{(LFSR)}$, n = 8.

Supplementary Fig. S2. Localization of HvPIP1;2 and HvPIP2;1 in *X. laevis* oocytes. (A) A fluorescent image of a sliced PIP1;2-expressing oocyte treated with anti-HvPIP1s antibody and anti-rat IgG (Alexa 647). Exposure: 0.55 sec, Bar: 100 μm. (B) Fluorescence intensity of (A) along the line indicated in the inset. (C) A fluorescent image of a sliced PIP2;1-expressing oocyte treated with the anti-HvPIP2;1 antibody and the anti-rabbit IgG (Alexa 488). Exposure: 0.002 sec, Bar: 100 μm. (D) Fluorescence intensity of (B) along the line indicated in the inset.

Supplementary Fig. S3. Amino acid alignment of the junction region of E-loop and transmembrane helix 6 of HvPIP2;1 to HvPIP2;5. Dagger indicates where I-254 of HvPIP2;3 and M-254 of HvPIP2;4 locate.

Supplementary Fig. S4. Alignment of partial sequences of deduced amino acids of PIP1 and PIP2 in *Arabidopsis thaliana*, *Oryza sativa*, *Zea mays* and *Hordeum vulgare*. The conserved isoleucine at the end of the E-loop is indicated by arrowhead, where M of HvPIP2;4 and V of ArPIP1;1 and AtPIP1;2 are exceptions. Accession number of maize PIP genes are Y243800 (ZmPIP1;1),

AF131201 (ZmPIP1;2), AF326487 (ZmPIP1;3), AF326488 (ZmPIP1;4), AF326489 (ZmPIP1;5), F326490 (ZmPIP1;6), AF326491 (ZmPIP2;1), AF326492 (ZmPIP2;2), AF326493 (ZmPIP2;3), F326494 (ZmPIP2;4), AF130975 (ZmPIP2:5), AF326495 (ZmPIP2;6) and AF326496 (ZmPIP2;7).

Supplementary Fig. S5. Effect of carbonic anhydrase (CA) on cytosolic acidification rate of *HvPIP2;1* cRNA-injected *X. laevis* oocytes. (A) Representative traces of pH decrease are shown. With CA, 25 ng carbonic anhydrase. Without CA, 0 ng carbonic anhydrase. Water, water-injected oocytes. HvPIP2;1, 50 ng *HvPIP2;1* cRNA-injected oocytes. Arrowheads indicate where the bath solution was replaced from 0.01 mM CO_2/H_2CO_3 to 6.5 mM CO_2/H_2CO_3. Magenta line indicates original pH of the cytosol. (B) 1/τ of *HvPIP2;1* cRNA-injected *X. laevis* oocytes. CA was co-injected with *HvPIP2;1* cRNA at indicated amount per oocyte. The acidification was initiated by replacing the bath solution from 0.01 mM CO_2/H_2CO_3 to 6.5 mM. Error bars indicate standard error. 0 ng CA, n = 2; 25 ng CA, n = 4; 50 ng CA, n = 3.

Supplementary Fig. S6. A typical calibration line of pH electrode. pH electrodes were calibrated as mentioned in Materials and Methods. $\Delta V = V_{pH} - V_r$, where V_{pH} and V_r indicate outputs of the hydrogen ion selective microelectrode and the membrane potential microelectrode, respectively. The slope was -61.5 mV pH^{-1} in the presented result. It was variable from -58.0 to -61.5 mV pH^{-1}, pipette to pipette.

Supplementary Tables

Supplementary Table S1. CO_2 permeability and the semi-conserved amino acid residue motif at the end of the E-loop of PIP2 members

Gene	Amino acid residues at the end of the E-loop	CO_2 permeability	Reference
HvPIP2;1	WIFWVGP	Yes	a
HvPIP2;2	WIFWVGP	Yes	a
HvPIP2;3	WIFWVGP	Yes	a
HvPIP2;4	WMFWVGP	No	a
HvPIP2;5	WIFWVGP	Yes	a
AtPIP1;2	WVFWVGP	Yes	b
AtPIP2;3	WIFWVGP	No	b
NtAQP1	WIFWVGP	Yes	b, c, d, e
NtPIP2;1	WIFWVGP	Yes/No	c, d
SsAqpZ	WLFWVGP	Yes	f

a, this study; b, Heckwolf et al. 2011; c, Uehlein et al. 2012b; d, Otto et al. 2010; e, Uehlein et al. 2003; f, Ding et al. 2013

Supplementary Table S2. Setting of the puller

	Heat	Pull	Velocity	Time	Pressure
Line 1	627	0	55	250	500
Line 2	627	0	30	250	
Line 3	627	0	50	250	

Supplementary Table S3. Primers for the construction of HvPIP2;3$^{(M254I)}$ and HvPIP;4$^{(I254M)}$

For HvPIP2;3$^{(I254M)}$	
23I254M_sen	gatgaccactggatgttctgggtggggc
23I254M_ant	gccccacccagaacatccagtggtcatc
For HvPIP2;4$^{(M254I)}$	
24M254I_sen	gatgaccactggatcttctgggtggggc
24M254I_ant	gccccacccagaagatccagtggtcatc

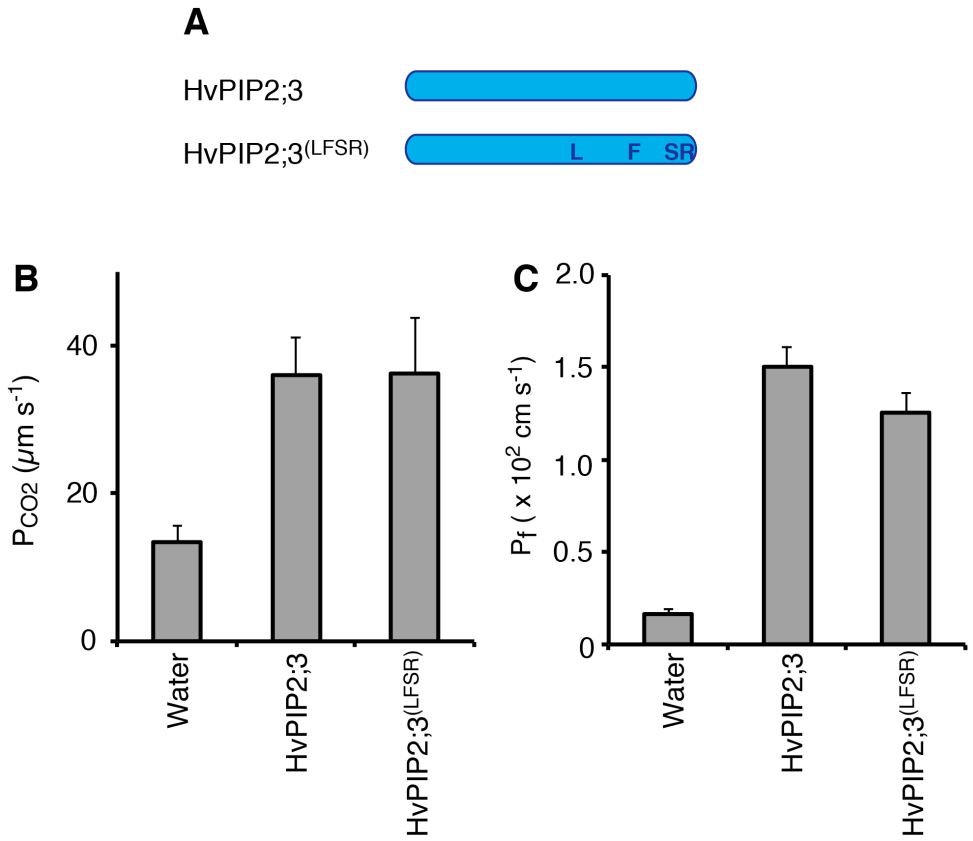

Supplementary Fig. S1. CO_2 permeability (P_{CO2}) of HvPIP2;3 with 4 amino acid substitutions at the carboxyl terminal stretch. (A) Illustrated presentation of HvPIP2;3$^{(LFSR)}$ construct. (B) P_{CO2} of *X. laevis* oocytes injected with water, *HvPIP2;3* cRNA and *HvPIP2;3*$^{(LFSR)}$ cRNA. Error bar indicates standard error. Water, n = 5; HvPIP2;3, n = 5; HvPIP2;3$^{(LFSR)}$, n = 4. (C) P_f of *X. laevis* oocytes injected with water, *HvPIP2;3* cRNA and *HvPIP2;3*$^{(LFSR)}$ cRNA. Error bar indicates standard error. Water, n = 8; HvPIP2;3, n = 9; HvPIP2;3$^{(LFSR)}$, n = 8.

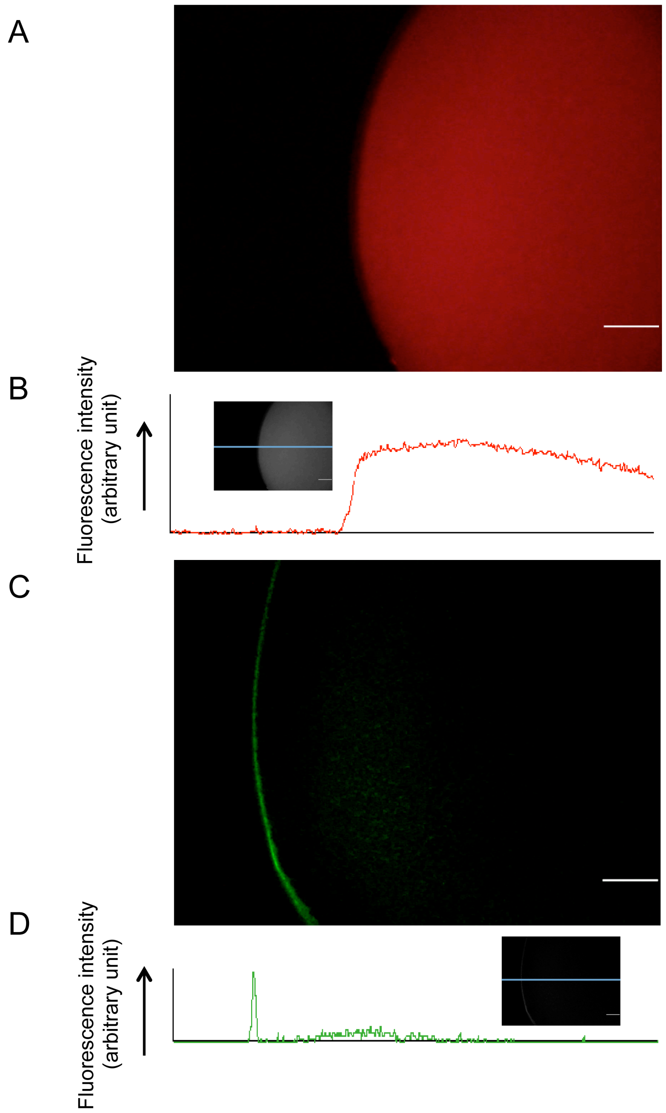

Supplementary Fig. S2. Localization of HvPIP1;2 and HvPIP2;1 in *X. laevis* oocytes. (A) A fluorescent image of a sliced PIP1;2-expressing oocyte treated with anti-HvPIP1s antibody and anti-rat IgG (Alexa 647). Exposure: 0.55 sec, Bar: 100 µm. (B) Fluorescence intensity of (A) along the line indicated in the inset. (C) A fluorescent image of a sliced PIP2;1-expressing oocyte treated with the anti-HvPIP2;1 antibody and the anti-rabbit IgG (Alexa 488). Exposure: 0.002 sec, Bar: 100 µm. (D) Fluorescence intensity of (B) along the line indicated in the inset.

```
                                   †
HvPIP2;1  AVIYNTDKAW DDQWIFWVGP LIGAAIAAAY
HvPIP2;2  AVIYNKKAAW DNHWIFWVGP FVGALAAAAY
HvPIP2;3  AVIYNNEKAW DDHWIFWVGP FIGAAIAAAY
HvPIP2;4  AVIYNNEKAW DDHWMFWVGP FIGAAIAALY
HvPIP2;5  AVIYNKDKAW DDQWIFWVGP MIGAAIAAFY
```

Supplementary Fig. S3. Amino acid alignment of the junction region of E-loop and transmembrane helix 6 of HvPIP2;1 to HvPIP2;5. Dagger indicates where I-254 of HvPIP2;3 and M-254 of HvPIP2;4 locate.

```
                                                                  ▼
AtPIP2_7        FAVFMVHLATIPITGTGINPARSFGAAVIYNN--EKAWDDQWIFWVGPFLGALAAAAYHQ
AtPIP2_8        FAVFMVHLATIPITGTGINPARSFGAAVIYNN--EKAWDDHWIFWVGPFVGALAAAAYHQ
OsPIP2_2        FAVFMVHLATIPITGTGINPARSIGAAVIYNQ--KKAWDDHWIFWAGPFIGALAAAAYHQ
HvPIP2_2        FAVFMVHLATIPITGTGINPARSLGAAVIYNK--KAAWDNHWIFWVGPFVGALAAAAYHQ
AtPIP2_2        FAVFMVHLATIPITGTGINPARSFGAAVIYNK--SKPWDDHWIFWVGPFIGAAIAAFYHQ
AtPIP2_3        FAVFMVHLATIPITGTGINPARSFGAAVIFNK--SKPWDDHWIFWVGPFIGATIAAFYHQ
AtPIP2_1        FAVFMVHLATIPITGTGINPARSFGAAVIYNK--SKPWDDHWIFWVGPFIGAAIAAFYHQ
AtPIP2_4        FAVFMVHLATIPITGTGINPARSFGAAVIYNN--EKAWDDQWIFWVGPMIGAAAAAFYHQ
AtPIP2_6        FSVFMVHLATIPITGTGINPARSFGAAVIYNN--QKAWDDQWIFWVGPFVGAAIAAFYHQ
AtPIP2_5        FAVFIVHLATIPITGTGINPARSLGAAIIYNK--DKAWDHHWIFWVGPFAGAAIAAFYHQ
OsPIP2_1        FAVFMVHLATIPITGTGINPARSLGTAVIYNK--DKAWDDQWIFWVGPLIGAAIAAAYHQ
HvPIP2_1        FAVFMVHLATIPITGTGINPARSLGAAVIYNT--DKAWDDQWIFWVGPLIGAAIAAAYHQ
ZmPIP2_3        FAVFMVHLATIPITGTGINPARSLGAAVIYNK--DKAWDDQWIFWVGPLIGAAIAAAYHQ
ZmPIP2_4        FAVFMVHLATIPITGTGINPARSLGAAVIYNK--DKAWDDQWIFWVGPLIGAAIAAAYHQ
OsPIP2_3        FAVFMVHLATIPITGTGINPARSLGAAVIYNQ--HKAWHDHWIFWVGPLIGAAIAAAYHQ
ZmPIP2_5        FAVFMVHLATIPITGTGINPARSLGAAVIYNN--DKAWDDHWIFWVGPFIGAAIAAAYHQ
HvPIP2_3        FAVFMVHLATIPITGTGINPARSFGAAVIYNN--EKAWDDHWIFWVGPFIGAAIAAAYHQ
HvPIP2_4        FAVFMVHLATIPITGTGINPARSFGAAVIYNN--EKAWDDHWMFWVGPFIGAAIAALYHQ
OsPIP2_4        FAVFMVHLATIPITGTGINPARSLGVAVVYNN--NKAWSDQWIFWVGPFIGAAIAALYHQ
ZmPIP2_6        FAVFMVHLATIPITGTGINPARSLGAAVVYNN--SKAWSDQWIFWVGPFIGAAIAALYHQ
OsPIP2_5        FAVFMVHLATIPVTGTGINPARSLGAAVVYNN--SKAWSDQWIFWVGPFIGAAIAALYHQ
ZmPIP2_1        FAVFMVHLATIPVTGTGINPARSLGAAVIYNK--DKPWDDHWIFWVGPLVGAAIAAFYHQ
ZmPIP2_2        FAVFMVHLATIPVTGTGINPARSLGAAVVYNK--DKPWDDHWIFWVGPLLGAAIAAFYHQ
OsPIP2_6        FAVFMVHLATIPITGTGINPARSIGAAVIFNN--EKAWHNHWIFWVGPFVGAAIAAFYHQ
HvPIP2_5        FAVFMVHLATIPITGTGINPARSLGAAVIYNK--DKAWDDQWIFWVGPMIGAAIAAFYHQ
ZmPIP2_7        FAVFMVHLATIPVTGTGINPARSFGPAVIFNN--DKAWDDQWIYWVGPFVGAAVAAIYHQ
OsPIP2_7        LAVLVVHLATIPITGTGINPARSLGPALVLGLGTTKAWSHLWIFWVGPFAGAAAAMIYHH
OsPIP2_8        FAVFVVHLATIPITGTGINPARSLGAAVLYNQ--HAAWKDHWIFWVGPVIGAFLAAAYHK
OsPIP2_9        FAVFVVHLATIPITGTGINPARSFGAAVVYNQ--PNAWHDQWIFWVGPLVGSAIATLYHE
AtPIP1_1        FAVFLVHLATIPITGTGINPARSLGAAIIYNK--DHSWDDHWVFWVGPFIGAALAALYHV
AtPIP1_2        FAVFLVHLATIPITGTGINPARSLGAAIIFNK--DNAWDDHWVFWVGPFIGAALAALYHV
AtPIP1_4        FAVFLVHLATIPITGTGINPARSLGAAIIYNK--DHSWDDHWIFWVGPFIGAALAALYHQ
AtPIP1_3        FAVFLVHLATIPITGTGINPARSLGAAIIYNK--DHAWDDHWIFWVGPFIGAALAALYHQ
AtPIP1_5        FAVFLVHLATIPITGTGINPARSLGAAIIYNK--DHAWDDHWIFWVGPFIGAALAALYHQ
ZmPIP1_3        FAVFLVHLATIPITGTGINPARSLGAAIIYNR--DHAWSDHWIFWVGPFIGAALAAIYHQ
ZmPIP1_4        FAVFLVHLATIPITGTGINPARSLGAAIIYNR--DHAWSDHWIFWVGPFIGAALAAIYHQ
ZmPIP1_2        FAVFLVHLATIPITGTGINPARSLGAAIIYNR--DHAWNDHWIFWVGPFIGAALAAIYHQ
OsPIP1_1        FAVFLVHLATIPITGTGINPARSLGAAIIYNK--DHAWNDHWIFWVGPFVGAALAAIYHQ
ZmPIP1_1        FAVFLVHLATIPITGTGINPARSLGAAVIYNQ--HHAWADHWIFWVGPFIGAALAAIYHQ
HvPIP1_5        FAVFLVHLATIPITGTGINPARSLGAAIIYNR--DHAWNDHWIFWVGPFVGAALAAVYHQ
HvPIP1_1        FAVFLVHLATIPITGTGINPARSLGAAIIYNR--EHAWSDHWIFWVGPFIGAALAAIYHQ
OsPIP1_2        FAVFLVHLATIPITGTGINPARSLGAAIVYNR--AHAWHDHWIFWVGPFIGAALAAIYHV
ZmPIP1_5        FAVFLVHLATIPITGTGINPARSLGAAIVYNR--SHAWNDHWIFWVGPFIGAALAAIYHV
HvPIP1_2        FAVFLVHLATIPITGTGINPARSLGAAIIYNK--KQSWDDHWIFWVGPFTGAALAAIYHV
HvPIP1_4        FAVFLVHLATIPITGTGINPARSLGAAIIYNK--KQAWDDHWIFWVGPFIGAALAAIYHV
HvPIP1_3        FAVFLVHLATIPITGTGINPARSLGAAIIYNK--KQAWDDHWIFWVGPFIGAALAAIYHV
ZmPIP1_6        FAVFLVHLATIPITGTGINPARSLGAAIIYDN--PHGWHGHWIFWVGPFAGAALAAVYHQ
OsPIP1_3        FAVFLVHLATIPITGTGINPARSLGAAIIYNR--GHAWDDHWIFWVGPFIGAALAAIYHQ
```

Supplementary Fig. S4. Alignment of partial sequences of deduced amino acids of PIP1 and PIP2 in *Arabidopsis thaliana*, *Oryza sativa*, *Zea mays* and *Hordeum vulgare*. The conserved isoleucine at the end of the E-loop is indicated by arrowhead, where M of HvPIP2;4 and V of ArPIP1;1 and AtPIP1;2 are exceptions. Accession number of maize PIP genes are Y243800 (ZmPIP1;1), AF131201 (ZmPIP1;2), AF326487 (ZmPIP1;3), AF326488 (ZmPIP1;4),

AF326489 (ZmPIP1;5), F326490 (ZmPIP1;6), AF326491 (ZmPIP2;1), AF326492 (ZmPIP2;2), AF326493 (ZmPIP2;3), F326494 (ZmPIP2;4), AF130975 (ZmPIP2:5), AF326495 (ZmPIP2;6) and AF326496 (ZmPIP2;7).

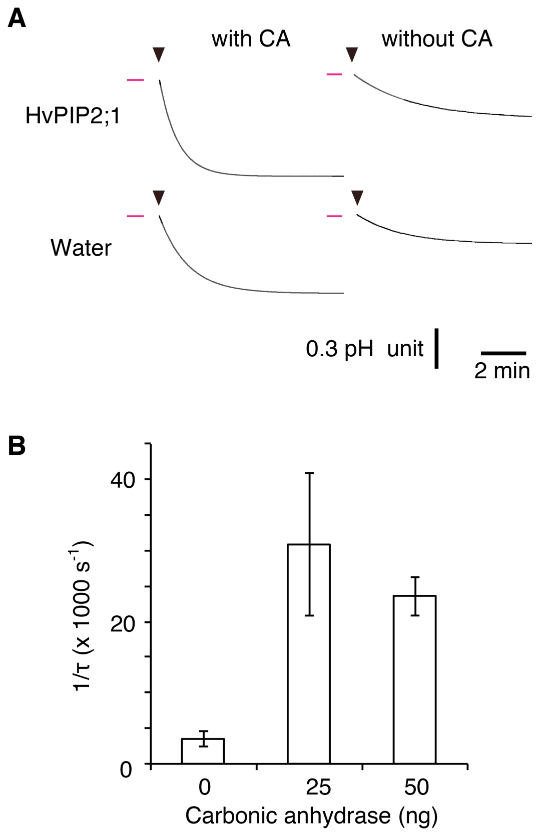

Supplementary Fig. S5. Effect of carbonic anhydrase (CA) on cytosolic acidification rate of *HvPIP2;1* cRNA-injected *X. laevis* oocytes. (A) Representative traces of pH decrease are shown. With CA, 25 ng carbonic anhydrase. Without CA, 0 ng carbonic anhydrase. Water, water-injected oocytes. HvPIP2;1, 50 ng *HvPIP2;1* cRNA-injected oocytes. Arrowheads indicate where the bath solution was replaced from 0.01 mM CO_2/H_2CO_3 to 6.5 mM CO_2/H_2CO_3. Magenta line indicates original pH of the cytosol. (B) 1/τ of *HvPIP2;1* cRNA-injected *X. laevis* oocytes. CA was co-injected with *HvPIP2;1* cRNA at indicated amount per oocyte. The acidification was initiated by replacing the bath solution from 0.01 mM CO_2/H_2CO_3 to 6.5 mM. Error bars indicate standard error. 0 ng CA, n = 2; 25 ng CA, n = 4; 50 ng CA, n = 3.

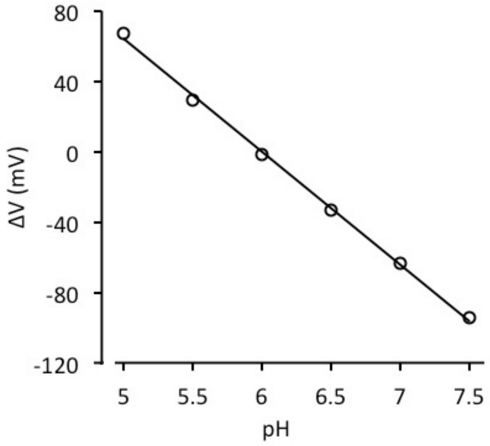

Supplementary Fig. S6. A typical calibration line of pH electrode. pH electrodes were calibrated as mentioned in Materials and Methods. $\Delta V = V_{pH} - V_r$, where V_{pH} and V_r indicate outputs of the hydrogen ion selective microelectrode and the membrane potential microelectrode, respectively. The slope was -61.5 mV pH^{-1} in the presented result. It was variable from -58.0 to -61.5 mV pH^{-1}, pipette to pipette.
